# Exploring the heterogeneous impacts of Indonesia’s conditional cash transfer scheme (PKH) on maternal health care utilisation using instrumental causal forests

**DOI:** 10.1007/s00181-025-02846-6

**Published:** 2026-02-12

**Authors:** Vishalie Shah, Julia Hatamyar, Taufik Hidayat, Noemi Kreif

**Affiliations:** 1 IQVIA UK, London, UK; 2 Centre for Health Economics, University of York, York, UK; 3 University of Sussex & Center for Health Economics and Policy Studies (CHEPS), Faculty of Public Health, Universitas Indonesia, Depok, Indonesia; 4 University of Washington, Seattle, WA USA

## Abstract

This paper uses instrumental causal forests, a novel machine learning method, to explore the treatment effect heterogeneity of Indonesia’s conditional cash transfer scheme on maternal health care utilisation. Using randomised programme assignment as an instrument for enrollment in the scheme, we estimate conditional local average treatment effects for four key outcomes: good assisted delivery, delivery in a health care facility, pre-natal visits, and post-natal visits. We find significant treatment effect heterogeneity by supply-side characteristics, even though supply-side readiness was taken into account during programme development. Mothers in areas with more doctors, nurses, and delivery assistants were more likely to benefit from the programme, in terms of increased rates of good assisted delivery outcome. We also find large differences in benefits according to indicators of household poverty and survey wave, reflecting the possible impact of changes in programme design in its later years. The impact on post-natal visits in 2013 displayed the largest heterogeneity among all outcomes, with some women *less* likely to attend post-natal check-ups after receiving the cash transfer in the long term.

## Introduction

In recent decades, conditional cash transfer (CCT) programmes have become a popular policy tool in many low- and middle-income countries for alleviating short-term poverty via cash injections, while also improving the long-term trajectory of vulnerable families via investments in human capital (Parker and Todd 2017). Regular cash payments are made to households in exchange for compliance with certain behaviours, such as school attendance for children, or attendance at health check-ups for new mothers, among others. Numerous evaluations of CCTs, mainly based on randomised experiments (for example, *PROGRESA* in Mexico and *PRAF* in Honduras), have demonstrated the ability of these interventions to make substantial improvements on education, consumption and health outcomes, particularly in the short-term (Fiszbein and Schady 2009; Millán et al. 2019; Bastagli et al. 2019; Owusu-Addo et al. 2018; García and Saavedra 2017; Lagarde et al. 2007; Kabeer and Waddington 2015).

The majority of the CCT evaluation literature to date has focused on average effects, with fewer studies formally analysing whether effects differ for population subgroups defined by observable characteristics. Anti-poverty programmes are expected to impact households differently depending on their ability to convert the cash injections into desirable outcomes, which is highly dependent on their own attributes (Ravallion 2005; Cooper et al. 2020). For example, urban mothers may already have easier access to preventive health care facilities to satisfy the health requirements for pre- and post-natal check-ups, compared to those in rural regions. The cash injection could assist rural households in addressing some of the financial barriers in accessing health care, such as transport costs. Understanding this type of heterogeneity in programme impacts can help to inform better policy targeting that, among other objectives, identifies households that are expected to benefit the most, and protects those that are expected to benefit the least (Cooper et al. 2020). Of those studies that do in fact explore subgroup effects for pre-specified populations, many find evidence of heterogeneity that is consistent with the broader literature suggesting that CCT effectiveness on health outcomes is modified through various social determinants of health, such as education, wealth and the urban-rural distinction (Owusu-Addo et al. 2018; Bastagli et al. 2019).

In this paper, we contribute to the growing evidence base on the heterogeneous effects of CCT programmes by evaluating Indonesia’s *Program Keluarga Harapan* (Family Hope Programme, or PKH) using a unique data set from a large-scale randomised experiment that was implemented in 2007 alongside a baseline survey and two follow-up surveys in 2009 and 2013. We are interested in exploring how enrolment into PKH has influenced maternal health care utilisation in the short-term (2009) and the longer-term (2013) by performing separate analyses for both time periods. Existing evaluations of PKH have focused on estimating overall average effects, finding notable improvements in various utilisation outcomes, such as the probability of having a facility delivery, or that the delivery is assisted by trained professionals (Kusuma et al. 2016; Cahyadi et al. 2020). Few studies have acknowledged that PKH impacts may be heterogeneous, and preliminary subgroup analyses that stratify treatment effects by pre-selected “effect modifiers” (e.g. gender, employment sector, parental education levels), have shown this to be the case (Kusuma et al. 2016; Alatas 2011). However, traditional approaches to heterogeneous treatment effect estimation (e.g. estimating treatment effects on effect modifier strata, or performing interactions between the treatment variable with effect modifiers in a linear regression) have their own limitations, including potentially arbitrary subgroup analyses and issues of multiple hypotheses testing. Recent developments in machine learning (ML)-based estimators of treatment effect heterogeneity use flexible modelling strategies that can capture complex interactions between theoretically-motivated covariates without imposing restrictive functional form assumptions. Generalised random forests, developed by Athey et al. (2019), have become a popular tree-based ML tool for estimating causal effects, including the conditional average treatment effect (CATE) function, which captures heterogeneity in treatment effects by flexibly modelling interactions among pre-selected effect modifiers informed by theory and prior empirical evidence.^1^

We rely on the random assignment of PKH to inform our empirical strategy. While the programme was randomised, actual enrolment was not random, due to non-compliance and targeted assignment within randomised populations. Using random assignment as an instrument for treatment status is a well-established approach in the literature on experimental evaluations with non-compliance (Angrist et al. 1993, 2006), as it addresses selection bias because the randomisation protocol is exogenous to potential outcomes. Following Triyana (2016) and Cahyadi et al. (2020), we address these potential observed and unobserved differences between enrolled and not enrolled groups using an instrumental variable analysis, where we instrument PKH enrolment with the original randomisation mechanism itself. While these previous studies focus on estimating average programme effects, we extend their analysis by estimating and characterising heterogeneous effects—revealing how impacts differ across population subgroups and identifying key drivers of this variation. We use instrumental causal forests; a variant of generalised random forests that allows for the presence of unmeasured confounding if there is a valid instrument available (Athey et al. 2019). This method targets the estimation of the so-called conditional local average treatment effect, characterising how treatment effects vary according to observed characteristics of compliers, in our case mothers who complied with the randomisation protocol. We summarise treatment effect heterogeneity using three approaches: (1) we find the best linear predictors of heterogeneity; (2) we assess how the most and least affected population groups differ in terms of observable characteristics, and (3) we estimate optimal policy trees of selected depth and describe which characteristics are chosen as the most important decision criteria for treatment allocation (Chernozhukov et al. 2018b; Semenova and Chernozhukov 2021; Knaus et al. 2021; Kennedy 2020; Athey and Wager 2019).

This paper has three main contributions. First, we add to the growing collection of CCT evaluation studies that look beyond average impacts and capture heterogenous impacts according to observable differences in covariates. A novel contribution is our use of data-driven methods, in particular tree-based causal ML, to estimate and make inferences on the heterogeneous impacts of a CCT intervention. Unlike previous heterogeneity evidence which tends to consistently report greater effects mostly among the poorer population, we find larger increases in facility delivery and post-natal visits for better-off households. Our findings could help to support those from existing heterogeneity analyses by identifying potentially new population subgroups that have not been specified in advance, such as groups defined by combinations of supply-side readiness, household poverty indicators, or geographic and temporal factors. By using data-driven methods, we allow for the discovery of interaction effects and subgroup patterns that may be overlooked in traditional stratified analyses. Second, to our knowledge, this is the first paper to evaluate a large-scale policy intervention using instrumental forests. Several published papers have used causal forests^2^ without incorporating an instrumental variable analysis to address endogeneity concerns (Kreif et al. 2022; Bertrand et al. 2017; Davis and Heller 2017; O’Neill and Weeks 2018; Hoffman and Mast 2019; Athey and Wager 2019). Third, by applying these methods to Indonesia’s national CCT programme, we aim to generate insights that can inform future programme design and targeting -particularly in relation to supply-side readiness and household-level characteristics that may shape programme effectiveness.

## The PKH programme

### Background and design

PKH was launched by the Government of Indonesia in 2007 as the country’s first CCT programme targeted to households. It was designed in response to increasing concerns around the country’s consistently poor human development outcomes (i.e. high mortality rates for new mothers and children under-5 and low enrolment rates for primary and secondary schools) compared to neighbouring countries, despite experiencing sustained economic growth. Prior to the implementation of PKH, an unconditional cash transfer programme (*Bantuan Langsung Tunai*, or BLT) was trialed but failed to achieve the desired outcomes due to ineffective targeting of the poor and a lack of conditions on the transfers to incentivise poverty-reducing behaviours (World Bank 2012). In comparison, PKH provides quarterly cash transfers to extremely poor households with pregnant women and/or children, with the objective of improving lagging health and education outcomes (Alatas 2011). The cash payments, ranging between 600,000 and 2,200,000 rupiah per quarter (approximately 60–330 US dollars, depending on household composition) were made to women in the household, who were informed at the start of the programme that in order to continue receiving payments, they must fulfil certain obligations. For example, pregnant or lactating women are required to make four pre-natal care visits and two post-natal visits, take iron tablets during pregnancy, and have an assisted delivery with a trained professional.^3^ The average duration of household enrolment into PKH is between 2 and 4 years, in which time the programme aims to achieve improvements in welfare and human development indicators.

The first phase of the PKH experiment was introduced in six provinces (West Java, East Java, North Sulawesi, Gorontalo, East Nusa Tenggara, and Jakarta). The selection of these provinces was based on willingness to participate and diversity representation of poverty levels, urban–rural characteristics, and remoteness. The richest 20 per cent of districts within each province were excluded. Then, 736 subdistricts that met minimum supply-side readiness criteria, corresponding to a population of about 36 million people, were identified. From this eligible pool, 438 subdistricts were randomly assigned to the treatment group, with the remaining subdistricts constituting the control group. Approximately 700,000 extremely poor households within these treated subdistricts were then enrolled as PKH participants via proxy means testing.

The World Bank collected data via a baseline survey in the months prior to launch, and two follow-up surveys in 2009 and 2013. Out of the 736 sampled subdistricts, 360 were randomly chosen for data collection (corresponding to approximately 14,000 households), which included beneficiary and non-beneficiary households in 180 treated subdistricts, and eligible households in 180 control subdistricts. To establish the stratification, the PKH sample considered urban and rural classification. The sampling frame was created by randomly selecting eight villages within each subdistrict, and then selecting one rural ward (*dusun*) within each village, or one urban precinct (*kelurahan*) within cities. Four households were randomly selected within each *dusun*/*kelurahan*, in a way that ensured two households included a pregnant or lactating mother or a married woman who was pregnant within the last 2 years, and the other two included children aged 6–15. The same households participated in the follow-up surveys which also used the original baseline questionnaire and respondent lists. The expansion of the programme post-2007 did not affect the composition of the control group to a large extent since new subdistricts, outside of the original sample, were prioritised for treatment. However, the value of the cash transfer fell from 14% of monthly household consumption in 2007 to 7% by 2013.

### Related literature

Existing evidence on the impacts of CCTs on health care utilisation is vast. Early impact evaluations of pioneering programmes implemented in Latin America and the Caribbean have generated substantial evidence on their effectiveness in increasing the utilisation of preventive health care services among the poor, and in some cases, improving health outcomes (Lagarde et al. 2007; Glassman et al. 2007; Ranganathan and Lagarde 2012). For example, there were substantial increases in pre-natal care visits of 8% and 19% in Mexico (*Progresa*) and Honduras (*Programe de Asignacion Familiar, PRAF* (Barber and Gertler 2009; Morris et al. 2004). Looking beyond average effects, Cooper et al. (2020) conducted a review into the existing literature reporting heterogeneity in programme impacts across population subgroups defined according to sex, socioeconomic status, region and education. Of the 56 reviewed studies, 40 reported subgroup effects presented either as stratum-specific effects or as interactions between effect modifiers and the intervention. Using evidence from India (*Janani Suraksha Yojana, JSY*) and Mexico (*Oportunidades*), they found that positive programme effects on health care utilisation were generally larger among women that are younger (aged 15–24), more disadvantaged, less educated, rurally-based, and from regions where the CCT scheme was more rigorously implemented.

In Indonesia, impact evaluations of PKH support these earlier findings that the cash incentives translate to greater health care demand. Cahyadi et al. (2020) find dramatic short- and longer-term effects of PKH on various behaviours (even after correcting for multiple hypothesis testing): an increase in the average number of post-natal visits (0.8) in 2009; and increases in the probability of having a facility delivery (17%) and a delivery assisted by a doctor or midwife (23%) in 2013. The authors, however, do not explore varying impacts across the population. Kusuma et al. (2016) similarly find encouraging effects on utilisation in 2009, including an increase in the proportion of women who had $\ge 4$ pre-natal visits (4%), $\ge$2 post-natal visits (5%), and a facility delivery (7%). They explore whether utilisation effects vary for pregnant women that are indicated as high-risk, finding that proportions of pre-natal visits and facility delivery decrease as risk increases. Alatas (2011) also finds substantial increases in the likelihood of beneficiary mothers completing $\ge 4$ pre-natal visits (13%) and $\ge 2$ post-natal visits (21%). They additionally reported subgroup effects, finding that PKH effects on newborn-related health care utilisation are greater among urban and non-agriculturally based households where health care facilities are more accessible and available, and among female-led households. Finally, they report that mothers with some formal education are more likely to have a facility delivery and make post-natal visits, whereas those with no education are more likely to have an assisted delivery.

### Data

**Fig. 1:**
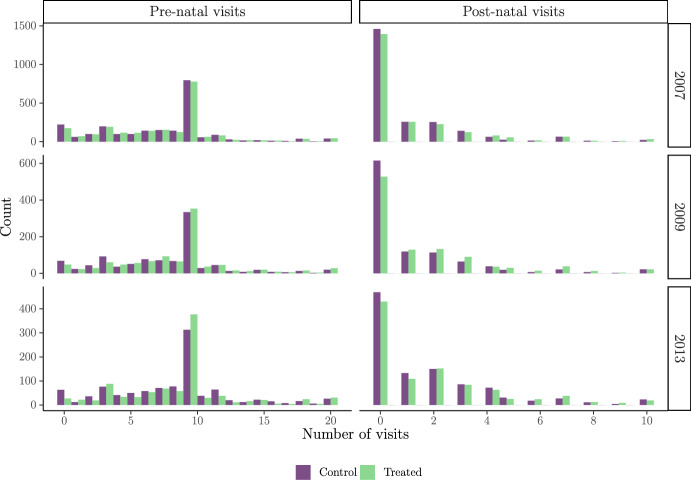
Distribution of pre- and post-natal health visits, by enrolment status and year. *Note*: The main analysis in this paper covers survey waves from 2009 and 2013, but here we present pre-experiment outcomes from 2007 as a basis of comparison

We construct a dataset of married women aged 16–49 who had pregnancies or deliveries within the 2 years prior to the 2009 and 2013 follow-up surveys. For the outcomes, we construct four binary variables related to health care utilisation that indicate whether the woman attended at least four pre-natal check-ups, the delivery took place at a medical facility, the delivery was assisted by a trained professional, and the woman attended at least two post-natal check-ups. We decide to discretise the continuous outcomes for the number of pre- and post-natal visits since PKH requires a specified minimum number of visits to be met in order to make the cash transfer. Figure 1 displays the distribution of the number of health visits made by control and treated populations both pre- and post-experiment.

We remove observations with incomplete data on the outcomes and the vector of covariates. This exclusion of observations could potentially introduce bias if the non-response to surveys is not random. However, as shown in Table 3, the characteristics of the randomly assigned treatment and control groups in our final analytical sample remain well-balanced, with all standardised mean differences (SMDs) below the 0.1 threshold. This suggests that sample attrition did not create systematic differences between the treatment and control groups. The final sample sizes for the main analysis are 2065 for 2009 and 1989 for 2013.

Table 1 presents the proportion of observations that comply with the randomisation protocol. In 2009, around half of those randomised to be in the programme were actually enrolled, with a non-negligible share of households—10% in 2009 14% by 2013—in control subdistricts enrolled in PKH. The presence of such ‘always-takers’ reflects non-compliance with the original randomisation protocol and the broader expansion of the programme beyond the initial experimental design. As PKH scaled nationally, some control areas received treatment due to administrative adjustments or targeting refinements.

Using the principal strata framework (Angrist and Imbens 1995; Angrist et al. 1996; Angrist 2004), we estimate that compliers made up 39.8% of the sample in 2009 and 34.4% in 2013. Always-takers accounted for 9.6% (2009) and 13.7% (2013), while never-takers accounted for 50.6% (2009) and 51.9% (2013).^4^ These shares indicate that compliers form a sizeable fraction of the population, suggesting that the LATE is informative for policy because it pertains to the marginal group induced into enrolment by program assignment (Angrist 2004).

**Table 1:** Contingency table showing the association between the randomisation protocol and enrolment status

2009		
(*N* = 2065)		
	Randomisation (*Z*)	
	Control	Treated
Enrolment (*D*)		
Not enrolled	929 (90%)	525 (51%)
Enrolled	99 (10%)	512 (49%)

2013		
(*N* = 1989)		
	Randomisation (*Z*)	
	Control	Treated
Enrolment (*D*)		
Not enrolled	884 (86%)	501 (52%)
Enrolled	140 (14%)	464 (48%)

Randomisation protocol (*Z*) indicates whether the mother lives in a treatment or control subdistrict. Enrolment status indicates whether the mother actually received PKH (“enrolled”) or not (“not enrolled”)

## Methods

### Estimation of treatment effects

We are interested in separately estimating the causal effects of being enrolled into the PKH programme (compared to not being enrolled) in 2009 and 2013 on various outcomes relating to maternal health care utilisation—the number of pre-natal visits, the number of post-natal visits, the probability of an assisted delivery by a skilled midwife or doctor, and the probability of delivery at a health facility. We perform these analyses separately, using a common notation *Y* for all outcomes, and *D* for the binary indicator of PKH enrolment. Although we use the general notation *Y* for outcomes, in our main analysis *Y* refers to binary indicators for each outcome, as described in Sect. 2.3.

Under the potential outcomes framework for causal inference, we denote the potential outcome that would be observed if individual *i* was enrolled into programme *d* by $Y_i(d)$. For motivation, we begin by defining two standard estimands: (1) the average treatment effect (ATE), which takes the expectation of the individual treatment effects across the population, $\tau =E[Y_i(1)-Y_i(0)]$; and (2) the conditional average treatment effect (CATE), which evaluates the ATE for individuals with the same covariate profile $X_i=x$, $\tau (x)=E[Y_i(1)-Y_i(0)|X_i=x]$. However, due to non-compliance in programme enrolment, the treatment $D_i$ is endogenous. We therefore adopt an instrumental variable (IV) approach, where the binary indicator $Z_i$-denoting whether the household is located in an initial PKH subdistrict–serves as an instrument for $D_i$. This setup identifies the Local Average Treatment Effect (LATE) and its conditional analogue, the Conditional Local Average Treatment Effect (CLATE), which represent the causal effect of treatment for compliers, conditional on covariates.

Following Athey et al. (2019), we define the relationship between $Y_i$ and $D_i$ using a structural model, $Y_i=m(X_i)+\tau (X_i)D_i+\varepsilon _i$, where $m(X_i)$ is a nuisance function whose shape is unspecified, and $\varepsilon _i$ is an error term. This model generalises the standard linear regression approach traditionally used for causal inference by allowing for flexible, data-driven estimation of both baseline outcome variation and heterogeneous treatment effects. The main distributional assumption is that the error term $\varepsilon _i$ is mean-independent of the treatment assignment conditional on covariates and instrument. Since PKH was targeted to households (and not randomly assigned) and there was some reported non-compliance, we cannot proceed with the assumption that $\varepsilon _i$ is independent of $D_i$. This means that a regression of $Y_i$ on $D_i$ will not yield a consistent estimate of $\tau (x)$. We, therefore, introduce an instrumental variable (IV) $Z_i$, which is a binary indicator for whether the household is located in an initial PKH subdistrict, and represents the study randomisation mechanism. If $Z_i$ has a causal effect on $D_i$ conditionally on $X_i=x$ (the “relevance” assumption), and affects $Y_i$ only through $D_i$ conditionally on $X_i$ (the “exclusion restriction”), then $\tau (x)$ can be identified as follows:

1 $$\begin{aligned} \tau (x) = \frac{\textrm{Cov}[Y,Z|X_i=x]}{\textrm{Cov}[D,Z|X_i=x]}, \end{aligned}$$

where the numerator is the average intention-to-treat effect for a subgroup defined by the covariate profile *x*, interpreted as the conditional effect of being given the opportunity to enrol into PKH, and the denominator is the share of compliers in the subgroup with covariate profile *x*. We can use heterogeneous treatment effect estimation methods that use the identification in (1) to estimate $\tau (x)$ as the conditional local average treatment effect (CLATE), by solving an estimation equation of the form:

2 $$\begin{aligned} E[\psi _{\tau (x),m(x)}(Y_i,D_i,Z_i)|X_i=x]=0 \;\text {for all} \; x\in \mathcal {X}, \end{aligned}$$

where,

3 $$\begin{aligned} \psi _{\tau (x),m(x)}= \left( \begin{array}{lr} Z_i(Y_i-D_i\tau (x)-m(x))\\ Y_i-D_i\tau (x)-m(x)\\ \end{array}\right) . \end{aligned}$$

Equality (2) encodes the identification assumptions required for consistent estimation: the first row in (3) corresponds to the condition that the instrument is uncorrelated with the error term, and the second row corrects to the condition that the error term has mean zero.

In the generalised random forest framework, an instrumental forest is defined to solve a weighted sample analogue of (2), where each observation *i* is assigned a weight $\alpha _i(x)$ that reflects its similarity to the target covariate profile *x*:

4 $$\begin{aligned} \sum _{i=1}^N \alpha _i(x) , \psi _{\tau (x),m(x)}(Y_i,D_i,Z_i) = 0, \end{aligned}$$

where *N* denotes the sample size. The weights $\alpha _i(x)$ are determined by the structure of the instrumental causal forests—described below—and represent the influence of each observation on the estimation of $\tau (x)$ at *x*. This weighting scheme defines local neighbourhoods where treatment effects are similar, allowing for flexibly estimating heterogeneity in treatment effects.

In practice, the instrumental causal forest is constructed using local two-stage least squares (2SLS) estimation. Compared to conventional 2SLS, this method accommodates complex interactions and nonlinearities in covariates. The approach also allows for the relationship between the instrument *Z* and treatment uptake *D* to be heterogenous across individuals, because the instrumental variable estimation is conducted locally among units with similar covariates. Consequently, the resulting CLATEs capture heterogeneity in both treatment effects and compliance behaviour, remaining local to the relevant complier subpopulations.^5^

Specifically, the local 2SLS regressions are performed within a small neighbourhood of observations—a leaf—using the residualised versions of the outcomes $Y_i - m(X_i)$, the treatment assignment $D_i - e(X_i)$ (where $e(x) = P[D|X_i]$ is the treatment propensity), and the instrument $Z_i - g(X_i)$ (where $g(x) = P[Z|X_i]$ is the instrument propensity), treatment effect estimates obtained by solving the local versions of the moment equations defined above.^6^,^7^ So-called instrumental (causal) trees (Athey et al. 2019) are formed by recursively partitioning the data into leaves in a way that maximises the within-leaf heterogeneity in treatment effects. The trees are constructed using a sample splitting technique referred to as “honesty”, to avoid overfitting (Athey et al. 2019). This procedure is repeated across many bootstrapped samples to limit noise arising from individual trees, with the tree ensemble representing the instrumental forest.

Individual treatment effects $\hat{\tau }(X_i)$ are estimated by evaluating $\hat{\tau }(x)$ at each covariate profile $X_i$. The treatment effects $\hat{\tau }(X_i)$ can also be aggregated over the entire population to provide an estimate of the local average treatment effect (LATE), by plugging in $\hat{\tau }(X_i)$ into a variant of the augmented inverse probability of treatment weighted estimator (also known as the doubly robust estimator), formed by taking the average of so-called doubly robust scores $\Gamma _i$:

5 $$\begin{aligned} \hat{\tau } = \frac{1}{N}\sum ^N_{i=1}\hat{\Gamma }_i,\quad \hat{\Gamma }_i = \hat{\tau }(X_i) + \frac{\left( \frac{Z_i-\hat{g}(X_i)}{\hat{g}(X_i)(1-\hat{g}(X_i))}\right) }{\delta (X_i)}(Y_i-\hat{m}(X_i)-(D_i-\hat{e}(X_i)\hat{\tau }(X_i)). \end{aligned}$$

Construction of this particular doubly robust score requires estimates of *m*(*x*), *e*(*x*) and *g*(*x*), which are separately estimated via regression forests. It also requires a so-called compliance score $\delta (X)=E[D|X,Z=1]-E[D|X,Z=0]$, which is an estimate of the causal effect of *Z* on *D* that is estimated via an auxiliary causal forest.^8^

### Inference on treatment effect heterogeneity

Once we have obtained estimates of the individual CLATEs and double robust scores for each individual, we can use these estimates to examine drivers of treatment effect heterogeneity in a data-driven way. One way to assess treatment effect heterogeneity is to perform a linear regression of the doubly robust scores $\Gamma _i$ on *X* to compare the relative contribution of covariates in predicting the CLATEs (Semenova and Chernozhukov 2021; Chernozhukov et al. 2018a). The resulting coefficients from the linear model are referred to as the best linear predictors (BLP) of CLATEs. These coefficients represent partial correlations conditional on a linear index of the other variables and should not be interpreted as causal effects. If a coefficient of the BLP for $X_i$ is positive and significant, we interpret as $X_i$ having a significant positive linear impact on the treatment effect heterogeneity, holding all other variables constant.^9^

Another way that Chernozhukov et al. (2018) suggest assessing treatment effect heterogeneity is through use of a “Classification Analysis” (CLANs). This involves partitioning data into quartiles according to the estimated double-robust scores $\Gamma _i$, in effect ranking the observations from low to high estimated treatment effects. For each effect modifier of interest, we regress the variable on the indicator of being in the most affected group, using ordinary least squares (OLS). This analysis is then repeated for the indicator of being in the least affected group. For each effect modifier, we test whether the difference between the two estimated coefficients is statistically significant. If the difference is significantly positive, then those individuals with characteristic $X_i$ experienced greater levels of the treatment effect. In contrast to the BLP analysis, this can be interpreted as a univariate analysis—as effect modifiers are analysed one by one, without controlling for the others—and can provide further evidence of treatment effect heterogeneity with respect to specific covariates expressed as binary indicators.

After examining the BLP and CLANs, we compute a variable importance measure. Variable importance (VI) in the context of causal forests quantifies the relative contribution of each covariate to the heterogeneity in treatment effects. This approach assesses how frequently a variable is used to split the data during the learning of the causal forest’s decision trees. Specifically, it is derived from the frequency with which each covariate is selected for splitting, weighted by the reduction in variance of the estimated conditional average treatment effects (CATEs) that these splits induce. This highlights covariates that are most influential in explaining heterogeneity in treatment responses during the model estimation process itself. We interpret these results with caution: there is emerging evidence that VI metrics obtained from causal forests and other causal machine learning algorithms can be biased if one of the (observed) confounding variables is also strongly involved in the treatment effect heterogeneity (Hines et al. 2022; Bénard and Josse 2023).^10^ However, as a qualitative approach, we can use the VI output as a comparison with the previous estimate-based BLP and CLAN results. We rank the top 10 variables according to their VI measure.

Finally, we turn to a method commonly used to learn optimal treatment allocation rules in an interpretable way: policy trees. The policy tree learning algorithm described by Athey and Wager (2021) performs exhaustive search over all possible trees using the estimated $\Gamma _i$, choosing as the final treatment rule the tree which maximises the overall treatment effect (Athey and Wager 2021). In other words, we estimate the optimal policy $\hat{\pi }(X_i)$ which maximises a value function $\hat{A_n}(\pi )$:

6 $$\begin{aligned} \hat{\pi }_n = \mathop {\textrm{argmax}}\left\{ \hat{A}_n(\pi ) : \pi \in \Pi _n\right\} ,\quad \hat{A}_n = \frac{1}{N}\sum ^N_{i=1}(2\pi (X_i)-1)\hat{\Gamma }_i, \end{aligned}$$

where $\Pi _n$ denotes the class of binary decision rules for sample size *N*. We are interested in whether those characteristics that show significant relationship to treatment effect heterogeneity using BLPs and CLANs are also most commonly used by the policy tree algorithm to assign treatment under the optimal policy $\hat{\pi }$. We report which variables are chosen as the most important decision criteria when assigning the optimal treatment regime. We consider depth-two and depth-three policy trees to ensure interpretability.^11^

**Table 2:** List of selected variables in *X*

Variable	Used in $\hat{m}(x)$
Enrolled into subsidised insurance	Yes
Enrolled into other (non-subsidised) insurance	Yes
Lives in Java	Yes
Lives in an urban area	Yes
Age (16–29; 30–39; 40–49)	Yes
Mother is educated (at elementary level)	Yes
Mother is employed	Yes
Head of household is educated (at elementary level)	Yes
Head of household is employed in agriculture sector	Yes
Head of household is employed in service sector	Yes
Household spends above average on alcohol and tobacco	Yes
Number of practising doctors in village per capita (q1–q3)	No
Number of practising nurses in village per capita (q1–q3)	No
Number of practising midwives in village per capita (q1–q3)	No
Number of practising traditional birthing attendants in village per capita (q1–q3)	No
Ln(size of household) (q1–q3)	Yes
Log(household non-food expenditure per capita) (q1–q3)	Yes
Household has no clean water	Yes
Household has no own latrine	Yes
Household has no septic tank	Yes
Household has no electricity	Yes
Village chief indicates “lack of healthcare facilities" a top 3 concern	No
Village chief indicates “lack of medical equipment" a top 3 concern	No
Village chief indicates “low healthcare awareness" a top 3 concern	No

Continuous variables have been discretised into terciles (q1–q3) to ensure overlap and create policy-relevant thresholds

The analytical steps taken in this paper are described as follows:

1. Train an instrumental forest with 2000 trees in the ensemble, tuning key hyperparameters (mtry, sample.fraction, honesty.fraction, and alpha) using cross-validation^12^ while maintaining standard defaults for other parameters:
  Nuisance parameters—*m*(*x*), *e*(*x*) and *g*(*x*)—are estimated using separate regression forests, where the propensity score *e*(*x*) is estimated without including supply side variables in *X*, since these variables are used to determine eligibility and sample construction (see Sect. 2.3).
  The entire covariate vector *X* is used for the recursive partitioning—see Table 2 for a list of covariates.
2. Predict $\hat{\tau }(X_i)$ by evaluating the trained instrumental forest for each observation’s covariate profile:
  Predictions are made “out-of-bag”, meaning that only the trees that did not use observation *i* during the training process are used in the prediction.^13^
3. Construct doubly robust scores $\hat{\Gamma }_i$ to compute the LATE $\tau$.
4. Assess the treatment effect heterogeneity captured by the forest outputs:
  Plot a histogram of the estimated CLATEs $\hat{\tau }(X_i)$.
  Perform a linear regression of $\hat{\Gamma }_i$ on $X_i$ to find the best linear predictors of the CLATEs, and plot the estimated regression coefficients.
  Test the difference between the most and least effected individuals for each variable (CLANs), and plot the estimated differences, with confidence intervals.
  Obtain the variable importance metric (VI) and rank the top ten most important variables used in the process of learning of the instrumental forest.
  Learn and plot a depth-two and depth-three policy tree to examine which covariates are chosen as most important splitting criteria.

We implement these steps for each outcome and year separately.

**Table 3:** Summary statistics

	2009 (*N* =2065)						2013 (*N* = 1989)					
	(*N* = 2065)						(*N* = 1989)					
	Randomisation			Enrolment			Randomisation			Enrolment		
	Control	Treated	SMD	Not enrolled	Enrolled	SMD	Control	Treated	SMD	Not enrolled	Enrolled	SMD
**Outcomes**												
Pre-natal visits $\ge 4$	0.644	0.700	0.056	0.677	0.661	−0.016	0.771	0.838	0.067	0.805	0.801	−0.004
Good assisted delivery	0.460	0.517	0.057	0.494	0.475	−0.020	0.721	0.768	0.047	0.755	0.719	−0.036
Facility delivery	0.778	0.846	0.067	0.803	0.835	0.032	0.817	0.838	0.021	0.838	0.805	−0.033
Post-natal visits$\ge 2$	0.286	0.366	0.080	0.309	0.368	0.059	0.412	0.441	0.029	0.436	0.404	−0.032
**Mother’s characteristics**												
Age:16–29	0.408	0.467	0.059	0.457	0.390	−0.068	0.537	0.578	0.041	0.570	0.528	−0.042
Age:30–39	0.488	0.436	−0.052	0.452	0.486	0.034	0.368	0.333	−0.036	0.351	0.351	0.000
Age:40–49	0.104	0.097	−0.007	0.091	0.124	0.034	0.095	0.089	−0.006	0.079	0.121	0.041
Educated (elementary)	0.802	0.828	0.027	0.818	0.809	−0.009	0.861	0.876	0.014	0.879	0.843	−0.037
Employed	0.215	0.200	−0.015	0.199	0.226	0.026	0.237	0.187	−0.051	0.209	0.220	0.011
Subsidized insurance	0.729	0.747	0.019	0.710	0.804	0.093	0.738	0.759	0.020	0.716	0.821	0.105
Other insurance	0.046	0.054	0.008	0.053	0.043	−0.010	0.050	0.062	0.012	0.059	0.048	−0.011
**Head of household’s characteristics**												
Educated(elementary)	0.696	0.728	0.033	0.710	0.715	0.005	0.775	0.779	0.004	0.791	0.747	−0.044
Works in agriculture	0.591	0.628	0.036	0.585	0.668	0.082	0.534	0.508	−0.026	0.510	0.548	0.038
Works in service sector	0.164	0.157	−0.007	0.162	0.159	−0.003	0.111	0.089	−0.022	0.108	0.083	−0.026
**Household characteristics**												
Lives in Java	0.611	0.621	0.010	0.662	0.506	−0.157	0.681	0.656	−0.025	0.725	0.540	−0.185
Urban location	0.129	0.115	−0.015	0.117	0.134	0.017	0.120	0.119	−0.001	0.115	0.131	0.016
Num. HH members (ln)	1.813	1.793	−0.061	1.796	1.821	0.075	1.817	1.818	0.003	1.804	1.850	0.132
Non-food exp (PC)	10.918	10.933	0.024	10.965	10.832	−0.204	11.521	11.569	0.072	11.581	11.459	−0.180
Alcohol/tobac exp (PC)	7.638	7.871	0.062	7.741	7.790	0.013	8.386	8.705	0.082	8.387	8.892	0.135
No clean water	0.869	0.891	0.022	0.881	0.877	−0.004	0.892	0.875	−0.017	0.886	0.877	−0.008
No latrine	0.483	0.505	0.022	0.460	0.576	0.116	0.456	0.452	−0.004	0.418	0.536	0.118
No septic tank	0.655	0.661	0.006	0.639	0.702	0.063	0.582	0.590	0.008	0.565	0.634	0.069
No electricity	0.226	0.202	−0.024	0.184	0.283	0.099	0.112	0.115	0.003	0.090	0.167	0.077
Health care supply (number of practising workers in village per capita*1000)												
Doctors	0.321	0.301	−0.025	0.301	0.334	0.043	0.266	0.259	−0.011	0.239	0.318	0.123
Nurses	0.591	0.585	−0.005	0.564	0.644	0.058	0.516	0.519	0.002	0.484	0.593	0.106
Midwives	0.523	0.518	−0.007	0.507	0.553	0.068	0.496	0.510	0.025	0.494	0.524	0.053
Trad. birth attendants	1.030	1.061	0.019	0.935	1.309	0.209	0.965	1.034	0.044	0.890	1.247	0.217
**Village characteristics (village head identified as top 3 concern)**												
Lack healthcare facilities	0.312	0.299	−0.013	0.309	0.298	−0.011	0.271	0.293	0.022	0.266	0.318	0.051
Lack medical equipment	0.170	0.211	0.041	0.175	0.229	0.054	0.159	0.193	0.034	0.170	0.189	0.019
Low health awareness	0.132	0.095	−0.037	0.115	0.111	−0.004	0.110	0.101	−0.010	0.103	0.113	0.010

All columns apart from SMD report covariate means. SMD = standardised mean difference. q1 = highest quantity. Observations with complete data included only

## Results

Table 3 presents summary statistics for our sample populations in 2009 and 2013. We report covariate means according by randomisation status *Z* (we refer to these as treated and control samples), defined as those living in subdistricts that are assigned versus not assigned to PKH. We also report covariate means for sample populations based on their actual enrolment status in the PKH, *D* (referring to these as enrolled and not enrolled samples). Overall, the table highlights that the target population is largely rural-based (approximately 90%) with household heads who work in agriculture, and that the majority (50–60%) live on the island of Java and lack household utilities such as running water.

As expected, the average characteristics for treated and control samples are similar given the random assignment of PKH to subdistricts, with none of the reported SMDs being greater than 0.1. When comparing enrolled and not enrolled mothers, we find some large differences, highlighting the socioeconomic disadvantages faced by enrolled mothers. For example, in both time periods, enrolled mothers are less likely to live in Java, have larger households, spend more on non-food items, and have a greater supply of traditional birth attendants in the village, compared to non-enrolled mothers. Between 2009 and 2013, we can observe an increase in non-compliance (only 77% of mothers living in subdistricts that are assigned to PKH were actually enrolled in 2013, compared to 84% in 2009 (see Table 1), leading to a further increase in the imbalance between enrolled and not enrolled mothers. This increased imbalance is most notable for supply-side variables, somewhat reducing the relative disadvantage of the enrolled group due to improved access to healthcare providers: we find that enrolled women live in villages that are more likely to have a greater supply of doctors and nurses per capita compared to non-enrolled women.

This descriptive information also allows us to contrast the characteristics of the compliers to the randomised population. The compliers—those who actually enrol into PKH—are more likely to be older (aged 30–49) and have subsidised health insurance. They are also more likely to be urban-based but less likely to live in Java, and they tend to have slightly larger households compared to the randomised population.

### Local average treatment effects

**Fig. 2:**
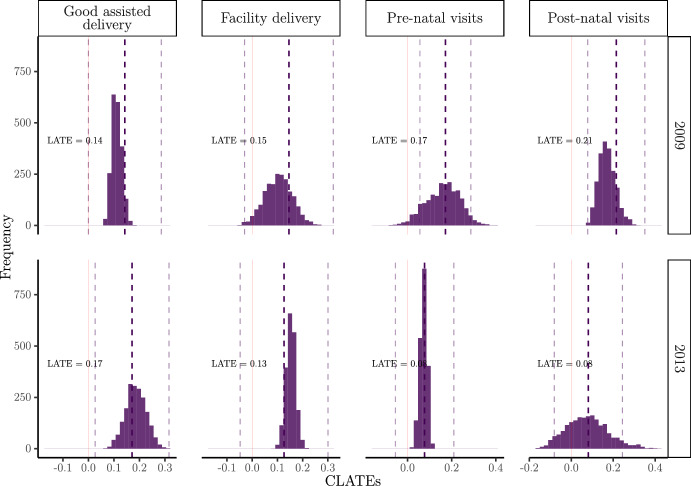
Histograms of estimated CLATEs $\hat{\tau }(x)$ from the instrumental forest, by outcome and year. *Note*: Dashed lines denote the ATE point estimates and 95% confidence intervals (via the Augmented Inverse Probability of Treatment Weighted (AIPTW) estimator). Red solid line at zero

Figure 2 displays histograms of the estimated CLATEs ($\hat{\tau }(x)$ from the tuned instrumental forest for each outcome and year).^14^ Looking firstly at local average effects, the programme has positive significant short- and longer-term impacts on the probability that the mother has a good assisted delivery (LATE = 0.14 (SE = 0.07) in 2009, LATE = 0.17 (SE = 0.07) in 2013). The beneficial average impacts on the probability that the mother meets the required threshold for pre- and post-natal visits are only significant in 2009 (pre-natal LATE = 0.17 (SE = 0.06), post-natal LATE = 0.21 (SE = 0.07)), suggesting that the programme has more immediate rather than sustained effects on health care visits.^15^ Lastly, we find no effect on the probability that the mother has a facility delivery in both time periods. To further validate our findings, we present numeric values for LATE estimates obtained from both the causal forest and a traditional two-stage least squares (2SLS) in Appendix Table 5. Estimates are largely consistent across the two models, with the exception that the 2SLS results are statistically significant for all outcomes except the pre-natal and post-natal visits in 2013.

Looking beyond average effects, the histogram in Fig. 2 provides evidence of treatment effect heterogeneity, but the extent and pattern of heterogeneity varies by outcome and year. For some outcomes, such as post-natal visits in 2013, the CLATEs span a wide range (from approximately −0.2 to 0.4), indicating substantial variation in individual treatment effects and suggesting that some compliers are less likely to attend post-natal check-ups after receiving the cash transfer. In contrast, for outcomes like good assisted delivery, the distributions of CLATEs are narrower, indicating less heterogeneity and fewer compliers displaying adverse behaviour in response to the programme. Not all outcomes show CLATE distributions that are symmetric or span both negative and positive values. For example, the distributions for good assisted delivery, facility delivery, and pre-natal visits are more concentrated and may not extend far into negative values, reflecting more consistent positive programme impacts for most compliers.

### Best linear predictors of treatment effects

**Fig. 3:**
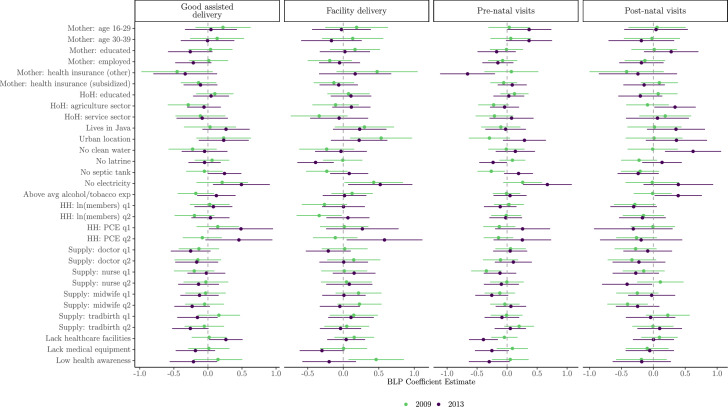
Estimated coefficients (and 95% CIs) from the best linear predictor analysis of $\Gamma _i$ on $X_i$. *Note*: Instrumental forest estimate of $\tau (x)$ *using the instrument* $Z_i$ *as treatment* $D_i$. HoH = head-of-household. HH = household. PCE = per capita expenditure. Tradbirth = traditional birth attendant. Continuous variables have been converted to discrete variables using terciles. Reference categories include: Age 40–49, Doctor:q3 (third tercile), Nurse:q3, Midwife:q3, Tradbirth:q3, HHsize:q3 and PCE:q3. q1 = largest quantity. Confidence intervals are obtained from the regression standard errors

Figures 3 and 8 present the estimated coefficients from the best linear predictor analysis, which linearly regresses the doubly robust scores $\hat{\Gamma }_i$ on $X_i$ for both binary and continuous outcomes. For the good assisted delivery outcome, the analysis shows that in 2009, having the head of household employed in the agriculture sector is associated with lower treatment effects, suggesting that the programme was less effective for these households. For agricultural households, this reduced effectiveness may reflect opportunity costs or different healthcare preferences in rural areas. In 2013, however, households lacking a septic tank or electricity, as well as those in villages where the chief identified “lack of healthcare facilities” as a primary concern, experienced higher treatment effects. This pattern indicates that PKH was particularly beneficial for the poorest households and those with limited access to health facilities.

Turning to facility delivery, the results for 2009 highlight that living in an urban area (but not Java) and village chief concern about “low health awareness” are strong positive predictors of increased treatment effects. In 2013, the only negative predictor identified is the lack of a household latrine, while lack of electricity remains a positive predictor. These findings suggest that the programme may have encouraged mothers who lacked appropriate provisions for home birth to seek facility-based deliveries. The temporal differences in facility delivery effects between 2009 and 2013 may reflect programme maturation effects and changing healthcare infrastructure over time.

For pre-natal visits, the 2009 results show that not having a septic tank and living in villages with the highest supply of nurses per capita are both associated with lower treatment effects. This may reflect that mothers already living in areas with better health worker supply did not change their health care demand in response to the programme. In 2013, the absence of household electricity is a positive predictor, while village chief concern about “lack of healthcare facilities” and enrollment in non-subsidised health insurance are negatively associated with treatment effects. These results point to the importance of travel time and facility access as barriers to programme effectiveness.

Finally, for post-natal visits, the analysis finds that being in the second highest tercile of midwives is a negative predictor in 2009. In 2013, being in the second tercile of nurses is associated with lower treatment effects, whereas households lacking clean water and heads of households working in agriculture are associated with higher treatment effects. This suggests that PKH was more effective for households with fewer amenities and less access to health personnel.

Notably, household per capita expenditure terciles show consistently small and mostly non-significant coefficients across outcomes and years, suggesting that within this targeted poor population, baseline wealth does not systematically predict treatment responsiveness. This indicates that expanding program eligibility to less poor households would be unlikely to yield systematically different returns. The patterns for the continuous outcomes are broadly similar, with lack of amenities and supply-side variables showing significant associations with treatment effect heterogeneity. Overall, these results reinforce the importance of both household-level deprivation and local health system readiness in shaping the impacts of the PKH programme.

### Classification analysis

**Fig. 4:**
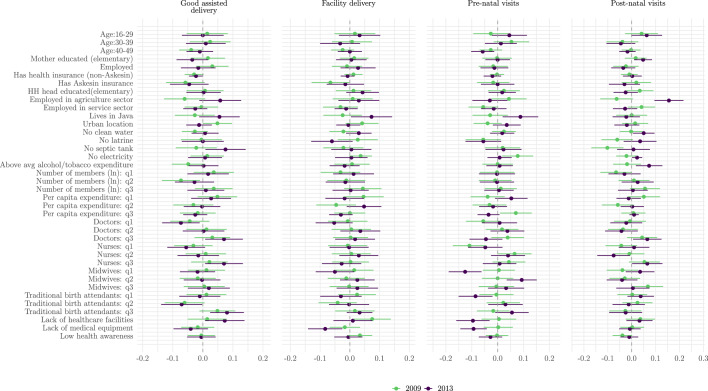
Mean differences (and 95% CIs) from the classification analysis (CLAN) of $\Gamma _i$.*Note*: Effect modifiers are regressed on indicators of being in the high or low treatment effect groups. HoH = head-of-household. HH = household. PCE = per capita expenditure. Tradbirth = traditional birth attendant. Continuous variables have been converted to discrete variables using terciles. Note this is a univariate analysis, and thus there are no reference categories. Confidence intervals are obtained from the regression standard errors

Figure 4 presents the results of the classification analysis (CLAN), which examines the univariate relationship between group membership and treatment effect for each effect modifier. In this analysis, a positive coefficient indicates that individuals in the group (for example, urban residents) experience higher treatment effects compared to those not in the group (such as rural residents), while a negative coefficient indicates lower treatment effects for the group relative to others.

For the assisted delivery outcome, the results show that urban residents had a higher treatment effect than rural residents in 2009, but this difference was not observed in 2013. Those without a septic tank experience higher levels of the treatment effect in 2009. In both years, the CLAN suggests that if the household has health insurance, but not public (Askeskin) insurance, there may be a lower treatment effect. For all of the supply-side variables (doctors, nurses, and midwives per capita), we see that those living with the lowest tercile had the highest treatment effects in 2013. For the facility delivery outcome, the results show that households without electricity had a higher treatment effect in 2009, but not 2013. The supply-side variables are less significant for this outcome. The households residing in Java were associated with higher levels of the treatment effect in 2013, and low village medical awareness was associated with lowest levels of the treatment effect for facility delivery in 2013.

**Table 4:** Top 10 Variable Importance for 2009 and 2013

Variable Ranking 2009		Variable Ranking 2013	
**Good assisted Delivery**			
Per capita expenditure: q2	0.05	Midwives: q3	0.0348
Age:30–39	0.0441	Lack of healthcare facilities	0.0336
No latrine	0.0414	No latrine	0.0332
Age:16–29	0.0399	Age:16–29	0.0331
Midwives: q1	0.0355	Traditional birth attendants: q1	0.0324
Midwives: q3	0.0351	Nurses: q1	0.0323
Number of members (ln): q3	0.0347	Doctors: q1	0.0311
Doctors: q1	0.0343	Per capita expenditure: q1	0.0302
Nurses: q3	0.0342	Number of members (ln): q3	0.03
HH head educated(elementary)	0.0338	No septic tank	0.0299
**Facility Delivery**			
HH head educated(elementary)	0.0319	No latrine	0.0468
Has Askesin insurance	0.0315	Employed in agriculture sector	0.0446
Doctors: q1	0.031	Number of members (ln): q2	0.0439
No latrine	0.0302	Age:16–29	0.0414
Employed	0.03	Nurses: q2	0.041
Per capita expenditure: q3	0.0295	Nurses: q1	0.0402
Employed in agriculture sector	0.0294	Midwives: q1	0.0396
Number of members (ln): q3	0.0293	No septic tank	0.0392
Midwives: q2	0.0292	Age:30–39	0.0392
Age:30–39	0.029	Number of members (ln): q3	0.0388
**Pre-natal visits**			
No electricity	0.0883	Employed in agriculture sector	0.0469
Nurses: q1	0.0852	No latrine	0.0447
Lack of healthcare facilities	0.0429	Number of members (ln): q2	0.0446
Per capita expenditure: q3	0.0396	Nurses: q2	0.0405
Nurses: q3	0.039	Age:16–29	0.0402
Employed	0.0373	Number of members (ln): q1	0.0398
Nurses: q2	0.0351	Age:30–39	0.0393
Midwives: q3	0.0306	Midwives: q1	0.0388
Mother educated (elementary)	0.0298	Nurses: q1	0.0386
**Post-natal visits**			
Lives in Java	0.0295	Number of members (ln): q3	0.0365
No septic tank	0.0511	Nurses: q3	0.0468
No latrine	0.05	Employed in agriculture sector	0.0413
Per capita expenditure: q2	0.0486	No clean water	0.0403
Age:16–29	0.0407	Doctors: q3	0.0374
Nurses: q1	0.0402	Doctors: q2	0.0347
Number of members (ln): q1	0.0401	Nurses: q2	0.0335
Midwives: q1	0.0397	Number of members (ln): q1	0.0328
Age:30–39	0.0383	Number of members (ln): q3	0.0327
Employed in agriculture sector	0.0378	Midwives: q2	0.0325
Doctors: q1	0.0377	Age:30–39	0.0316

Numeric values obtained from the GRF variable_importance *function for causal forests*

For the pre-natal visits outcome, we see major differences in the CLAN results between survey waves. In 2013, for example, many of the supply variables (highest terciles of nurses and midwives, lack of facilities and medical equipment) are associated with low levels of the treatment effect, in contrast to what we observe for 2009. We also observe that living in Java is associated with higher levels of heterogeneous treatment effects for pre-natal visits in 2013, suggesting that long-term incentives for these visits may be more effective in regions with adequate transportation infrastructure. For the post-natal visits outcome, we see that households without a septic tank have lower levels of the treatment effect, as do those employed in the agriculture sector in 2009. However, those in the agriculture sector experience significantly higher levels of the treatment effect in 2013, as do those living in the lowest terciles of nurses and doctors (but highest tercile of midwives). These results could be explained by differences in the type of personnel most likely to be present for a pre- or post-natal visit (doctor or nurse vs midwive) according to geographic characteristics. Across both analysis methods, we observe no clear socioeconomic gradient in treatment effects. Variables reflecting household wealth and socioeconomic status show mixed and generally non-significant patterns, contrasting with the consistent importance of healthcare supply variables.

### Variable importance

**Fig. 5:**
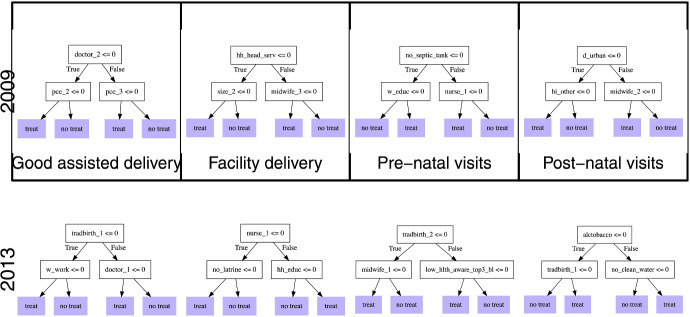
Depth-two policy trees learned from $\Gamma _i$. *Note*: The top row presents trees for 2009, and the bottom row for 2013. Each column depicts one of the four outcomes. w_educ:“Mother educated”,w_work:“Mother employed”, hh_head_agr:“HoH agriculture sector”,hh_head_serv:“HoH service sector”, d_java:“Lives in Java”,d_urban:“Urban location”, no_clean_water:“No clean water”, no_latrine:“No latrine”, no_septic_tank:“No septic tank”, no_electricity:“No electricity”, alctobacco:“Above avg alcohol/tobacco exp”, pce_1:“HH PCE q1”,pce_2:‘HH PCE q2”, pce_3:“HH PCE q3”, Supply: doctor_1:“doctor q1”, doctor_2:“doctor q2”, doctor_3:“doctor q3”, nurse_1:“nurse q1”, nurse_2:“nurse q2”, nurse_3:“nurse q3”, midwife_1:“midwife q1”, midwife_2:“midwife q2”, midwife_3:“midwife q3”, tradbirth_1:“tradbirth q1”, tradbirth_2:“tradbirth q2”, tradbirth_3:“tradbirth q3”, tradbirth_4:“tradbirth q4”, low_hlth_aware_top3_bl:“Low health awareness”

Next, we assess the variable importance results presented in Table 4. These results are largely consistent with the findings of the BLP and CLAN analyses, though we interpret them with caution. Note that the actual numeric value obtained from the variable importance metric is meaningless, and only the relative ranking of this metric is used in qualitative analysis. For the assisted delivery outcome in both years, the supply-side variables are four of the top 10 importance measures. In 2013, for example, the lowest tercile of midwives was ranked as the top most important variable. In both years, lack of a household latrine is the third most important variable. For facility delivery, the lack of a latrine remains important, and is the most important variable in 2013, followed by employment in the agriculture sector. For the pre-natal visits outcome, lack of household electricity is the most important variable in 2009, and employment in the agriculture is the most important in 2013. These variables are associated with living in urban areas and lack of healthcare facilities, which were identified as important in the previous BLP and CLAN results. The highest number of nurses per-capita is ranked as the second most important variable for the pre-natal visits outcome, similar to the BLP results. For post-natal visits, again, living in Java and lack of septic tank or latrine were important in 2009, whilst having a lower number of family members, lowest tercile of nurses and being employed in the agriculture sector were the most important variables in 2013 (See Fig. 5).

Overall, household characteristics associated with poverty (no electricity, latrine, or septic tank), as well as the supply-side readiness variables, are often listed as highly important across all years and outcomes. We note that household education, per-capita expenditure, insurance status, number of household members, and maternal age are also often included in the top ten important variable ranking lists, though they did not appear in the earlier analyses. These are likely variables that are highly correlated with the other household aspects, and although they are used in the learning and splitting of the causal forest trees, and therefore listed as important here, they are not picked up in the BLP or CLAN results, which rely on information from the individual treatment effect estimates themselves.

### Policy trees

We first examine the depth-2 decision trees learned from the estimated double robust scores $\hat{\Gamma }_i$. The trees are depicted in Fig. 9, with each row representing a survey year and each column representing one of the four outcomes under analysis. Each node of the decision trees presents a condition upon which the next choice of node relies. For example, in the 2009 assisted delivery tree, the first node condition is that the supply of doctors is not in the second tercile ($\le 0$). If this condition is true, then the following node condition is that the household is not in the second consumption expenditure tercile. If that condition is true, then the algorithm assigns treatment, and if not, treatment is not assigned. In all eight decision trees, there is an importance of healthcare worker supply in terms of decision criteria. For 2013, the top decision node in three of the four outcomes is related to health worker supply. For the good assisted delivery outcome, for example, the first decision criteria is whether there are a high number of traditional birth attendants, and the right bottom node depends on whether there is a high supply of doctors.

There are major differences in learned trees between the two survey waves. In 2009, the most important decision criteria for all four outcomes included mother’s education and health insurance status, the household per-capita expenditure, size, urban location, and lack of septic tank, as well as supply of all types of healthcare personnel. In 2013, there is slightly less influence of the mother and household characteristics on the decision rules, with the nodes being for maternal employment, lack of latrine, electricity or clean water, and household head education and alcohol/tobacco expenditure. All four health worker supply variables were important splitting criteria for the optimal treatment allocation.

**Fig. 6:**
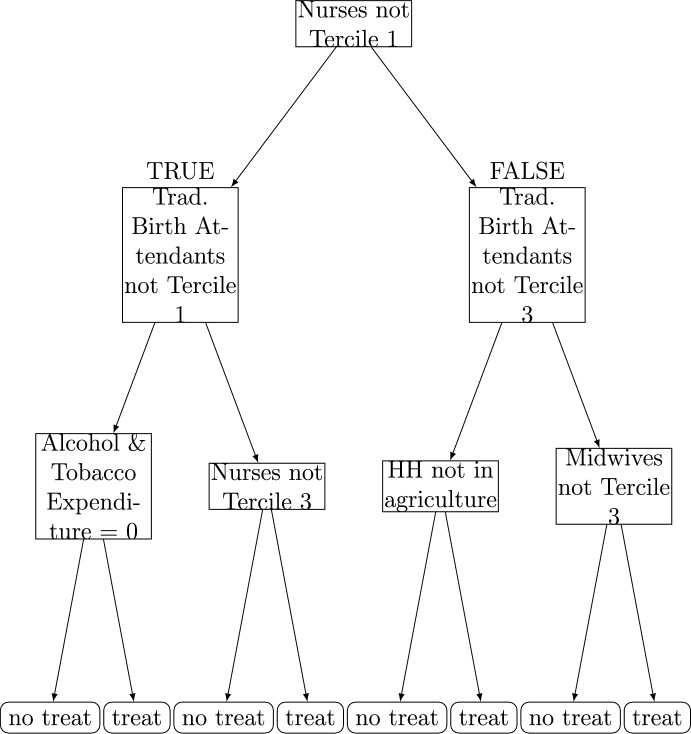
Depth-3 policy tree, Post-Natal Visits, 2013

**Fig. 8:**
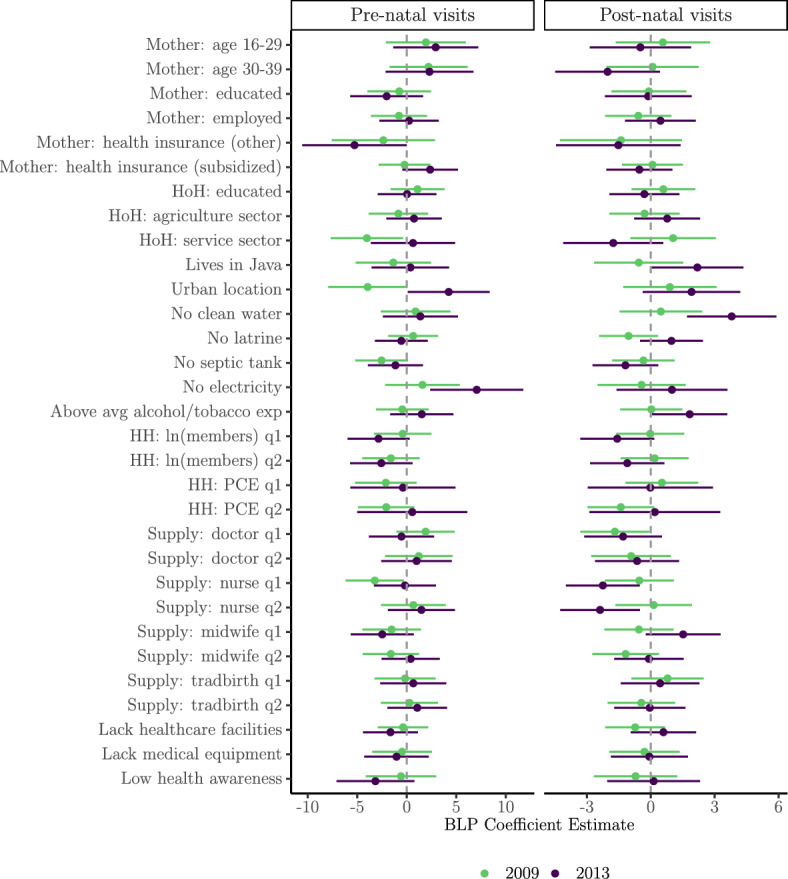
Estimated coefficients (and 95% CIs) from the best linear predictor analysis of $\Gamma _i$ on $X_i$. *Note*: Instrumental forest estimate of $\tau (x)$ *using the instrument* $Z_i$ *as treatment* $D_i$. HoH = head-of-household. HH = household. PCE = per capita expenditure. Tradbirth = traditional birth attendant. Continuous variables have been converted to discrete variables using quintiles. Reference categories include: Age 40–49, Doctor:q4, Nurse:q4, Midwife:q4, Tradbirth:q4, HHsize:q4 and PCE:q4. q1 = largest quantity

**Fig. 9:**
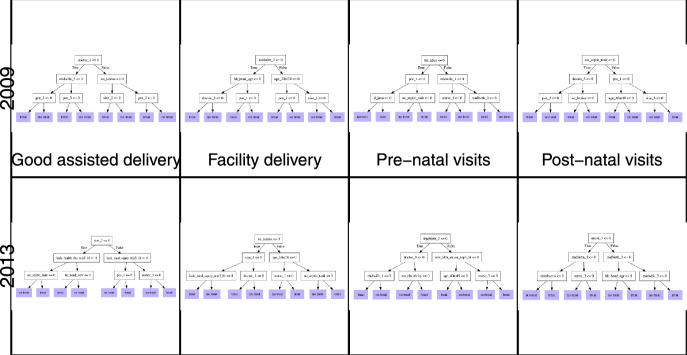
Depth-three policy trees learned from $\Gamma _i$. *Note*: The top row presents trees for 2009, and the bottom row for 2013. Each column depicts one of the four outcomes. w_educ:“Mother educated”,w_work:“Mother employed”, hh_head_agr:“HoH agriculture sector”,hh_head_serv:“HoH service sector”, d_java:“Lives in Java”,d_urban:“Urban location”, no_clean_water:“No clean water”, no_latrine:“No latrine”, no_septic_tank:“No septic tank”, no_electricity:“No electricity”, alctobacco:“Above avg alcohol/tobacco exp”, pce_1:“HH PCE q1”,pce_2:‘HH PCE q2”, pce_3:“HH PCE q3”, Supply: doctor_1:“doctor q1”, doctor_2:“doctor q2”, doctor_3:“doctor q3”, nurse_1:“nurse q1”, nurse_2:“nurse q2”, nurse_3:“nurse q3”, midwife_1:“midwife q1”, midwife_2:“midwife q2”, midwife_3:“midwife q3”, tradbirth_1:“tradbirth q1”, tradbirth_2:“tradbirth q2”, tradbirth_3:“tradbirth q3”, tradbirth_4:“tradbirth q4”, low_hlth_aware_top3_bl:“Low health awareness”, lack_health_fac_top3_bl:“Lacks healthcare facilities”, lack_med_fac_equip_bl:“Lacks medical equipment”

Finally, as a lower-depth decision tree will pick up more granular treatment assignment rules, we also examine depth-3 policy trees. Here we focus on the depth-3 tree for the post-natal visit outcome in 2013, which exhibits a very wide distribution of estimated CATEs and also a large portion of that distribution with negatively estimated (harmful) treatment effects.^16^ For this depth-3 tree (Fig. 6), the first node relies on the supply of nurses, and both the second depth nodes rely on the supply of traditional birth attendants. The third layer of the tree sees some influence of household characteristics (alcohol and tobacco expenditure and whether the head of household works in agriculture), with the remaining nodes relying on healthcare supply. Overall, this deeper tree may better reflect the influence of the supply of nurses, birth attendants and midwives on whether the programme effectively incentivised post-natal visits. Depth 3 trees for all outcomes and years are reported in the Appendix.

Taken together and examined qualitatively, the policy tree results indicate a strong influence of the supply-side readiness of each village in terms of maximising desired maternal healthcare demand. The results also suggest that effects are important for those poorer households in urban locations and with fewer household amenities, indicating that the PKH programme was successful in incentivising those poorest participants. For example, in 2013 the bottom left decision node for the post-natal visits outcome assigns treatment to those who do not have clean water (no clean water $\le 0$ is false $\rightarrow$ treat). The inclusion of household consumption-expenditure in nodes of the deeper trees for 2009 aligns with the variable importance findings.

## Discussion

In this paper, we used data on new mothers from a randomised experiment to evaluate the local average and heterogeneous effects of the PKH programme on various maternal health care utilisation outcomes in 2009 and 2013. We used a causal machine learning method, instrumental forests, to estimate heterogenous treatment effects (CLATEs), and aggregated these estimates over the entire sample population to produce a doubly robust approximation to the LATE.^17^ We also performed three types of complementary analysis of the drivers of heterogenous treatment effects: explored the best linear predictors of treatment effects, conducted a classification analysis, and built interpretable policy trees.

Our results largely support those from early evaluations on the overall average impacts of PKH on (compliant) new mothers, with increases in the probabilities of having a good assisted delivery in 2009 and 2013, attending at least four pre-natal check-ups in 2009, and attending at least two post-natal check-ups in 2009. However, the sizes of the effects tend to vary across studies, which can be explained by variations in study designs resulting in differences in covariate selection and identification of causal effects. Beyond average effects, the distribution of CLATEs provides evidence of heterogeneity in treatment effects such that although most mothers are expected to increase health care demand in response to the cash transfer based on their observed characteristics, others are less affected.

We acknowledge limitations of our analysis. Although a covariate balance check did not point to systematic differences between the treatment and control group, any imbalance on unobserved covariates would lead to possible attrition bias. In this work, we define treatment as a binary indicator for enrolment into PKH, and estimate conditional local average treatment effects accordingly. While the size of the cash transfer varies across households based on composition and eligibility, we do not model the treatment as continuous. Instead, we include covariates that influence transfer size as part of the forest estimation, allowing us to indirectly account for variation in programme generosity. A more direct approach would involve treating the transfer amount as a continuous treatment and estimating dose-response functions. This would require a different identification strategy, and is therefore beyond the scope of this paper. We leave this as a promising avenue for future research.

Previous evidence shows the supply of health care is the main source of regional variations and has a positive association with health care utilization (Finkelstein et al. 2016; Godøy and Huitfeldt 2020; Skinner 2011). To increase healthcare access by removing the demand-side barrier, using policies like the CCT programme, supply-side improvements are important to consider (Ensor and Cooper 2004; Gertler 2004; Gruber et al. 2014; Triyana 2016). Our analysis of drivers of treatment effect heterogeneity suggests that location and supply-side factors are important determinants of varying treatment effects for several outcomes. Urban-based households, where health care supply is more readily available, due to better proximity of medical facilities and a greater supply of practicing health care workers, are less likely to change their demand for maternal health care in response to the cash transfer. Other related variables, such as whether the household is located in Java and the nature of employment of the head-of-household, which is inherently linked to geographical factors, are also identified to be important predictors. We find that for the most part, the estimated regression coefficients from the BLP and the CLAN analysis are significant for one time period only, either 2009 or 2013, with only a few maintaining their significance throughout both periods, indicating a changing role of characteristics in programme effectiveness over time.

Our study may provide some insights into the factors affecting the duration and distribution of policy effects. The finding that PKH is unable to consistently maintain effectiveness beyond the short-term, if at all, could be explained by some reported issues in programme design and implementation (Kusuma et al. 2016). Administrative problems resulting in payment delays and missed payments altogether could partly explain the limited impact, combined with the fact that cash payments (as a proportion of household consumption) were essentially halved between 2007 and 2013, thus significantly reducing the incentive-based component of the policy. Our results also suggest that geographical factors that are inherently linked to health care supply are the primary drivers of treatment effect heterogeneity, rather than socioeconomic differences within the target population. The absence of a clear wealth gradient in treatment effects supports the program’s targeting strategy of focusing on extremely poor households in supply-ready areas, as expanding to less poor populations would unlikely yield systematically greater benefits. Instead, ensuring adequate healthcare infrastructure in program areas appears crucial for maximising effectiveness among intended beneficiaries. Although PKH aims to target poor households in supply-ready areas, residual differences in health care accessibility and availability seem to contribute to varying policy impacts. It has been argued that, in addition to supply-side readiness, other contextual differences, including cultural factors and supply-side barriers, can impact programme effectiveness (Glassman et al. 2013). For example, poor quality of care, transportation costs and a lack of health knowledge or programme awareness may restrict health care use irrespective of the value of the cash payment or the availability of health facilities in the local area (Gaarder et al. 2010). These findings suggest the need to better align demand-side policies with supply-side initiatives to support policy effectiveness.

## Figures and Tables

**Fig. 7:**
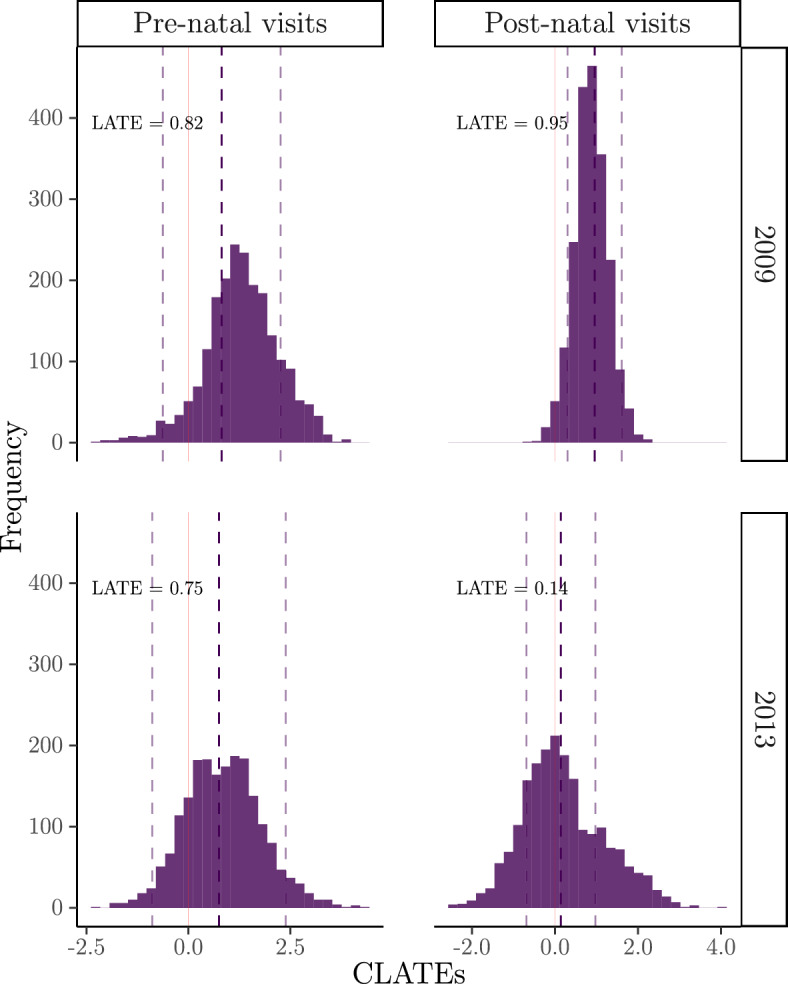
Histograms of estimated CLATEs $\hat{\tau }(x)$ from the instrumental forest, by outcome and year. *Note*: Dashed lines denote the ATE point estimates and 95% confidence intervals (via the AIPTW estimator). Red solid line at zero

## Estimates

See Tables 5 and 6.

**Table 5:** ATEs and standard errors, all outcomes

Year	Outcome	Causal Forest		2SLS	
		LATE	se	LATE	se
2009	Good assisted delivery	0.14	0.07	0.13	0.05
2009	Facility delivery	0.15	0.09	0.14	0.05
2009	Pre-natal visits	0.17	0.06	0.15	0.04
2009	Post-natal visits	0.21	0.07	0.18	0.05
2013	Good assisted delivery	0.17	0.07	0.20	0.05
2013	Facility delivery	0.13	0.09	0.15	0.06
2013	Pre-natal visits	0.08	0.07	0.04	0.05
2013	Post-natal visits	0.08	0.08	0.11	0.06

**Table 6:** Estimated compliance shares

	2009	2013
**Angrist & Imbens** (principal strata framework)		
Compliers	39.8%	34.4%
Always-takers	9.6%	13.7%
Never-takers	50.6%	51.9%
**Athey** (grf)		
Compliers	38.8%	33.8%

## Robustness checks

### Continuous version of pre/post-natal visits outcomes

See Figs. 7, 8 and 9.

## Footnotes

1 For an overview of the different causal machine learners of heterogeneity parameters, we refer the reader to Lechner and Mareckova (2024).

2 Causal forests, as referred to in this paper, are specifically those developed within the generalised random forest (GRF) framework by Athey and Wager (2019). We acknowledge that other causal forest approaches exist which rely on different estimation principles (e.g., Lechner and Mareckova 2022; Cockx et al. 2023)

3 The programme also required that children receive immunizations and attend school.

4 Using a separate causal forest to obtain an estimated compliance score for each year (i.e., the first stage of the instrumental forest), we find estimated compliance of 38.8% in 2009 and 33.8% in 2014, consistent with the principal strata estimates. We also report these values in the Appendix.

5 For full details on the estimation procedure and its theoretical properties, we refer readers to Athey et al. (2019)

6 Following Robinson (1988), residualisation helps to mimimise confounding bias due to observed covariates by partialling out the effects of $X_i$.

7 Note that *m*(*x*), *e*(*x*) and *g*(*x*) are collectively referred to as the “nuisance parameters” since they are not primarily of interest but are required to estimate the target causal parameter. In an instrumental forest, the nuisance parameters are internally estimated using separate regression forests, a predictive machine learning algorithm.

8 Causal forests also rely on the generalised random forest framework but find neighbourhoods of observations where the CATEs are similar. Note that when the instrument *Z* and treatment *D* are the same, an instrumental forest is equivalent to a causal forest.

9 To ensure valid inference, we rely on out-of-bag predictions from the instrumental forest, which means that the doubly robust scores are computed independently of the observations used in the regression step (see Athey et al. 2019). This avoids overfitting and supports valid inference without requiring an additional sample split. While cross-fitting or explicit sample splitting for the BLP analysis would offer stronger theoretical guarantees (see Chernozhukov et al. 2018b), we opted not to implement these due to the limited sample size.

10 Variable importance measures may also favour continuous or highly variable covariates, which is addressed in this work by the discretising of continuous covariates into terciles.

11 Practical application of the policy tree framework to an IV setting may face a conceptual challenge. While policy trees combined with instrumental variable (IV) estimates can assist in describing heterogeneity, we note that their interpretability is strictly limited to compliers (whose identity remains unobserved), thus complicating inference for non-compliers and defiers. Takatsu et al. (2025) provide methods to profile compliance types in using double robust IV methods, and the application of these methods in a policy tree framework is subject to future work.

12 We tuned four key hyperparameters using the built-in parameter tuning option in the grf package: mtry (number of variables sampled at each split), sample.fraction (fraction of observations sampled), honesty.fraction (fraction used for treatment effect estimation), and alpha (maximum split imbalance). The ensemble size was set to the standard default of 2000 trees. The tune.num.trees parameter (200 trees) controls the size of auxiliary forests used during hyperparameter optimisation rather than the final ensemble size. For complete methodological details on parameter selection and forest construction, see Athey et al. (2019).

13 Out-of-bag prediction produces CLATE estimates without the need for an additional data splitting step (Athey et al. 2019). Standard errors of the predicted CLATEs are clustered at the subdistrict level; the level of randomisation.

14 Point estimates and standard errors are reported in the Appendix.

15 Appendix Figure B.7 presents the distribution of the estimated CLATEs for the continuous versions of the health visits outcomes. The tuned instrumental forest analysis finds a significant positive effect for post-natal visits in 2009 (LATE = 1.04, SE = 0.33), but does not find significant effects for the continuous versions of pre-natal visits in either year, nor for post-natal visits in 2013.

16 We obtain the deeper trees using the fastpolicytree R package of Cussens et al. (2025)

17 Although compliance is imperfect, the complier group constitutes a sizeable fraction of the population, roughly 35–40%. This suggests that our LATE estimates are policy-relevant, as they capture the effect for those marginal households induced to participate by program assignment. Nonetheless, because always- and never-takers are unaffected by assignment, caution is warranted in generalising the LATE to the full population (Heckman 1997; Deaton 2010)
